# Combination of TLR2 and TLR3 agonists derepress infectious bursal disease virus vaccine-induced immunosuppression in the chicken

**DOI:** 10.1038/s41598-019-44578-5

**Published:** 2019-06-03

**Authors:** Khalid Bashir, Deepthi Kappala, Yogendra Singh, Javeed Ahmad Dar, Asok Kumar Mariappan, Ajay Kumar, Narayanan Krishnaswamy, Sohini Dey, Madhan Mohan Chellappa, Tapas Kumar Goswami, Vivek Kumar Gupta, Saravanan Ramakrishnan

**Affiliations:** 1 Immunology Section, ICAR - Indian Veterinary Research Institute, Izatnagar, Bareilly, Uttar Pradesh (243 122) India; 2 Division of Pathology, ICAR - Indian Veterinary Research Institute, Izatnagar, Bareilly, Uttar Pradesh (243 122) India; 3 Division of Animal Biochemistry, ICAR - Indian Veterinary Research Institute, Izatnagar, Bareilly, Uttar Pradesh (243 122) India; 4 Division of Animal Reproduction, ICAR - Indian Veterinary Research Institute, Izatnagar, Bareilly, Uttar Pradesh (243 122) India; 5 Division of Veterinary Biotechnology, ICAR - Indian Veterinary Research Institute, Izatnagar, Bareilly, Uttar Pradesh (243 122) India; 6 Centre for Animal Disease Research and Diagnosis, ICAR - Indian Veterinary Research Institute, Izatnagar, Bareilly, Uttar Pradesh (243 122) India

**Keywords:** Toll-like receptors, Adjuvants

## Abstract

Live intermediate plus infectious bursal disease virus (IBDV) vaccines (hot vaccines) are used for protection against the virulent IBDV strains in young chickens. We evaluated the potential of Toll-like receptor (TLR) agonists to alleviate hot vaccine-induced immunosuppression. The combination of Pam3CSK4 and poly I:C synergistically upregulated *IFN-β*, *IFN-γ*, *IL-12*, *IL-4*, and *IL-13* transcripts and cross-inhibited *IL-1β*, *IL-10*, and *iNOS* transcripts in the chicken peripheral blood mononuclear cells (PBMCs) as analyzed by quantitative real-time PCR. Further, four-week old specific pathogen free White Leghorn chickens (*n* = 60) were randomly divided into six groups and either immunized with hot IBDV vaccine with or without Pam3CSK4 and/or poly I:C or not vaccinated to serve as controls. The results indicated that poly I:C alone and in combination with Pam3CSK4 alleviated vaccine-induced immunosuppression, as evidenced by greater weight gain, increased overall antibody responses to both sheep erythrocytes and live infectious bronchitis virus vaccine, upregulated *IFN-γ* transcripts and nitric oxide production by PBMCs (*P < *0.05), and lower bursal lesion score in the experimental birds. In conclusion, poly I:C alone and its combination with Pam3CSK4 reduced the destruction of B cells as well as bursal damage with restoration of function of T cells and macrophages when used with a hot IBDV vaccine.

## Introduction

Gumboro disease is caused by infectious bursal disease virus (IBDV), which belongs to the genus *Avibirnavirus*, family *Birnaviridae*. IBDV is highly contagious and resistant to the common disinfectants. It contains a bisegmented double stranded RNA genome and undergoes frequent mutations that results in the generation of new variants. IBD has been of great concern for the past three decades^1,2^. The Organization for Animal Health (OIE) listed this disease as a high-morbidity and high-mortality causing disease that predisposes the birds to secondary infections^1^. IBDV targets the bursa of Fabricius (BF) in 3-6-week-old chicks resulting in immunosuppression, predisposing the chicks to opportunistic pathogens and leading to poor immune responses to subsequent vaccines^3–5^. There are two serotypes of IBDV, and serotype 1 is pathogenic^6^.

Vaccination is the main tool to control and prevent IBD, as biosecurity measures alone are insufficient. Live IBDV vaccines are preferred as they mimic natural infection, replicate inside the host and induce both cellular and humoral immunity. Different modified live IBDV vaccines have been developed and are classified as “mild,” “intermediate,” and “intermediate plus” vaccines, depending upon their ability to stimulate immunity despite the presence of maternally derived antibodies that can neutralize vaccine viruses^7^. Intermediate plus IBD vaccines (hot vaccines) are used mainly in IBD endemic regions where infection pressure is high and young chickens tend to have variable but often high maternally derived antibody titers. The modified live vaccines, while stimulating specific immunity, induce immunosuppression reducing the response to subsequent vaccinations due to bursal damage^8–10^. In addition, IBDV infection decreases the number of B cell clones and macrophages, impairs the phagocytic capacity of macrophages and reduces the ability of T cells to respond to mitogenic stimulation^11^.

Various Toll-like receptor (TLR) agonists have been explored as adjuvants and prophylactic agents for use in mammals and chicken. The TLR2 agonist, lipoteichoic acid, inhibited the replication of infectious laryngotracheitis virus in chicken embryos when administered 24 h prior to infection^12^. The TLR3 agonist, polyinosinic: polycytidylic (poly I:C) increased the expression of *IFN-γ*, *IL-6* and *IL-12* when used as an adjuvant with avian influenza virus^13^. Earlier, we reported the enhancement of antigen-specific humoral and cellular immune responses as well as protection against infection with the virulent Newcastle disease (ND) virus, when R-848, a TLR7 agonist was used with an inactivated ND vaccine in specific pathogen free (SPF) chickens^14^. Recently, we reported the enhancement of antigen-specific systemic and mucosal immune responses when R-848 was used with avian infectious bronchitis virus vaccines^15^, as well as the prophylactic potential of R-848 against infection with very virulent IBDV in SPF chickens^16^. Although a single TLR agonist is capable of inducing potent immune responses, a combination of TLR agonists might minimize the dose and side effects, mimic the natural infection and induce more balanced or desirable immune responses. The combination of TLR agonists can result in additive, synergistic, or even antagonistic immune responses. Synergy occurs between TLRs located in different regions of the cell, *viz*., surface or endosomal^17^. Although the effects of TLR2 and TLR3 interactions in immune cells of mice and humans have been examined, they have not been explored in chickens. Hence, in the present study, we aimed to evaluate the immunomodulatory effect of Pam3CSK4 (a synthetic triacylated lipopeptide, TLR2 agonist) and/or poly I:C (a TLR3 agonist) on live intermediate plus IBDV vaccine-induced immunosuppression in chickens.

## Materials and Methods

### Experimental birds

Embryonated eggs (*n* = 80) of SPF White Leghorn chickens were procured from Venky’s India Private Limited, Pune, India and hatched at Central Avian Research Institute, Izatnagar, India. Birds were maintained following standard management practices and provided with autoclaved feed and water *ad libitum*. The experiment was approved by the Institute Animal Ethics Committee (IAEC), Indian Veterinary Research Institute, Izatnagar, Bareilly, Uttar Pradesh, India 243122 (Approval letter No. F. 26-1/2015–16/J.D (R) dated October 25, 2016). All the animal experiments were carried out following the guidelines and regulations of IAEC.

### TLR2 and TLR3 agonists

TLR2 agonist, Pam3CSK4 (InvivoGen, CA, USA.) and TLR3 agonist, poly I:C (Sigma, MO, USA) were dissolved in sterile nuclease- and endotoxin-free water.

### Vaccines and enzyme linked immunosorbent assay (ELISA) kits

Commercially available live intermediate plus IBDV (chick embryo-adapted IBDV serotype 1, Venky’s India Private Limited, Pune, India) and infectious bronchitis virus (IBV) vaccines (Massachusetts strain, Venky’s India Private Limited, Pune, India) were procured. ELISA kits for IBDV and avian infectious bronchitis virus (IBV) were procured from IDEXX Laboratories, USA.

### Experiment 1: Effect of Pam3CSK4 and/or poly I:C on chicken peripheral blood mononuclear cells (PBMCs) *in vitro*

#### Chicken PBMC isolation and stimulation

Blood was collected from 2-week old SPF birds (*n* = 6) in syringes containing heparin (20 IU/mL) and was transferred to 15 mLcentrifuge tubes containing an equal volume of Ficoll-Histopaque 1.077 (Sigma, MO, USA) to separate PBMCs aseptically by density gradient centrifugation. The cells were washed twice with phosphate-buffered saline (PBS, pH 7.2) and re-suspended in RPMI-1640 complete medium containing 2% fetal bovine serum and 100 IU/mLpenicillin and 100 µg/mL streptomycin. Cell viability was determined by the trypan blue dye exclusion method and the cell count was adjusted to 1 × 10^7^ cells/mL. The PBMCs were stimulated at 40 °C and 5% CO_2_ with Pam3CSK4 and/or poly I:C at 10 and 50 µg/mL, respectively, as reported elsewhere by adding to the cell media^18–20^. The cells were harvested at 0, 3, 12, 24 and 48 h post-stimulation (*n* = 6/group/time point) to determine the relative expression of immune response genes.

#### Isolation of total RNA from PBMCs

Cells were pelleted by low speed centrifugation (2000 × *g*) for 5 min, and 750 µL RiboZol (Amresco, USA) was added. Chloroform (250 µL) was added to this mixture and vortexed for 30 s. The mixture was centrifuged at 12,000 × *g* for 30 min at 4 °C for phase separation. The aqueous phase rich supernatant with RNA was transferred to a new RNase-free microcentrifuge tube. Isopropanol (0.8 volumes) was added and centrifuged at 12,000 × *g* for 20 min at 4 °C. Supernatant was discarded without disturbing the RNA pellet. The pellet was washed with 500 µL of 70% ethanol by centrifugation at 12,000 × *g* for 15 min at 4 °C and air dried by inverting the tube on a clean filter paper for about 10 min to remove traces of ethanol. Finally, the pellet was dissolved in 20 µL RNase-free water and stored at −20 °C until further use. The purity of RNA was checked by measuring the absorbance at 260 and 280 nm with a Nanodrop UV spectrophotometer (Thermo Scientific, USA).

#### Preparation of complementary DNA (cDNA)

Total RNA isolated from PBMCs was used to prepare cDNA employing a RevertAid First Strand cDNA Synthesis Kit (Thermo Scientific, USA) following manufacturer’s instructions. Briefly, 2 µg total RNA and 1 µL of random hexamer (Thermo Scientific, USA) were added to nuclease-free water to reach a volume of 12.5 µL and incubated at 65 °C for 5 min. Then, the following reagents were added: 5× reaction buffer (4 µL), Ribolock RNase inhibitor (0.5 µL), 10 mM dNTP mix (2 µL), and RevertAid reverse transcriptase (1 µL). Tubes were mixed gently by vortexing, incubated at 25 °C for 10 min followed by 50 °C for 50 min for cDNA synthesis and the reaction was terminated by heating at 85 °C for 5 min. The cDNA product was stored at −20 °C until further use.

#### Quantitative real-Time PCR

Expression of *IL-1β*, *IFN-β*, *IFN-γ*, *IL-12*, *IL-4*, *IL-13*, *IL-10* and *iNOS* transcripts was analyzed by real-time PCR using a QuantiFast SYBR Green qPCR kit (Qiagen, CA, USA) on a CFX 96 Real-Time System (Bio-Rad, CA, USA). Previously published gene specific primers were used (Table 1), and the housekeeping gene β-actin was used to normalize expression levels^21^. The real-time PCR mixture consisted of 2 µL cDNA, 10 µL QuantiFast SYBR Green Master Mix, primers (0.5 µL each, 10 pmol concentration), and nuclease-free water to a volume of 20 µL. Real time PCR was performed using the following program: first cycle at 95 °C for 5 min, followed by 40 cycles each of 94 °C for 30 s, 60 °C for 45 s, 70 °C for 45 s and a final cycle of  94 °C for 30 s. The final step was to obtain a melting curve for determining amplification specificity. Each sample was run in duplicate on the same plate. The difference in cycle threshold (ΔCt) values for the target and β-actin gene was calculated. The delta Ct of the unstimulated control group at 0 h served as a calibrator to calculate the relative fold change of the target genes in other treated groups using the 2^−ΔΔCt^ method^22^.

**Table 1:** Primers used for quantitative real time PCR.

Target Gene	Primer sequence (5′-3′)	Product size (bp)	Reference
β-Actin	F: TATGTGCAAGGCCGGTTTC	110	^89^
	R: TGTCTTTCTGGCCCATACCAA		
IL-1β	F: GGATTCTGAGCACACCACAGT	272	^89^
	R: TCTGGTTGATGTCGAAGATGTC		
IFN-β	F: GCTCACCTCAGCATCAACAA	187	^89^
	R: GGGTGTTGAGACGTTTGGAT		
IFN-γ	F: TGAGCCAGATTGTTTCGATG	152	^89^
	R: CTTGGCCAGGTCCATGATA		
1L-12	F: CGAAGTGAAGGAGTTCCCAGAT	123	^90^
	R: GACCGTATCATTTGCCCATTG		
IL-4	F: GGAGAGCATCCGGATAGTGA	186	^89^
	R: TGACGCATGTTGAGGAAGAG		
IL-13	F:CTGCCCTTGCTCTCCTCTGT	123	^90^
	R:CCTGCACTCCTCTGTTGAGCTT		
IL-10	F: GCTGAGGGTGAAGTTTGAGGAA	142	^90^
	R: GAAGCGCAGCATCTCTGACA		
iNOS	F: AGGCCAAACATCCTGGAGGTC	371	^89^
	R: TCATAGAGACGCTGCTGCCAG		

#### Nitric oxide (NO) estimation

The PBMCs (1 × 10^7^ cells/mL) collected from the SPF chickens (*n* = 6/group) were resuspended in RPMI 1640 medium containing l-arginine (5 mM) and were stimulated with Pam3CSK4 (10 µg/mL) and poly I:C (50 µg/mL), individually and in combination. After stimulation, the cells were incubated for 24 and 48 h at 37 °C in 5% CO_2_ concentration. The quantity of NO was measured by the nitrite in the supernatant using Griess assay as previously described^23^. Briefly, Griess reagent (Sigma, MO, USA) 50 µL was added to 50 µL of sample and incubated at 37 °C for 30 min. The optical density was measured at 550 nm with a spectrophotometer. The standard curve used to interpolate the concentration of nitrite in the samples was constructed with different concentrations of sodium nitrite.

### Experiment 2: Evaluation of immunomodulatory activity of Pam3CSK4 and/or poly I:C administered with live intermediate plus IBDV vaccine

#### Immunization plan

Four-week-old SPF chickens (*n* = 60) were allotted randomly to one of six groups (*n* = 10/group) as shown in the Table 2. Pam3CSK4 and poly I:C were administered through intra-muscular route, whereas the IBDV vaccine was administered by ocular instillation.

**Table 2:** Immunization plan in the experimental birds.

Group	Treatment^$^		
	Commercial Live intermediate plus IBDV vaccine	Pam3CSK4 (20 μg/bird)	Poly I:C (200 μg/bird)
A (PBS control)	−	−	−
B (Vaccine alone)	+	−	−
C (Pam3CSK4 group)	+	+	−
D (Poly I:C group)	+	−	+
E (Combination group)	+	+	+
F^$$^ (Combination in half dose group)	+	+	+

^$ + ^Indicates addition while – indicates no addition.

^$$^Half dose of Pam3CSK4 (10 μg/bird) and poly I:C (100 μg/bird) was used.

IBDV: Infectious bursal disease virus.

#### Evaluation of the humoral immune response against IBDV

Blood samples were collected (*n* = 6/group) for serum separation at 7, 14, and 21 days post-IBDV immunization (dpi). The sera were stored at −20 °C until they were used to assay humoral immune response against IBDV using IDEXX ELISA kit according to the manufacturer’s instructions.

### Effect of TLR agonist(s) on IBDV vaccine-induced immunosuppression

Immunosuppression induced by live intermediate plus IBDV vaccine administered to the experimental birds was evaluated by assessing the following parameters: humoral responses against sheep red blood cells (sRBCs) and IBV vaccine, *IFN-γ* expression, macrophage function, histopathology of bursae, bursa to body weight (B/B) ratio. Weight gain in the experimental birds was also evaluated.

#### Humoral response against sRBCs and IBV vaccine

To evaluate post-IBD vaccination antibody responses, the experimental birds in groups B-F (*n* = 7/group) were immunized with sRBCs (10% v/v at a dose rate of 1 mL/bird, intramuscular route) and live IBV vaccine (ocular instillation) at 21 dpi^24^. A booster was given 14 days later, and the humoral response was measured at weekly intervals until 56 dpi. Birds in the group A were divided into two subgroups to serve as positive and negative controls. Both sRBCs and live IBV vaccine were administered to positive control birds (*n* = 3), whereas PBS was introduced intramuscularly as a negative control (*n* = 4).

Humoral response against sRBCs: Immune responses against sRBCs (*n* = 6/group; except the negative control and positive control, with *n* = 3/group) were assessed by hemagglutination (HA) test. Briefly, PBS (0.05 mL) was dispensed into each well of a 96-well plastic V-bottomed microtiter plate. In the first well (A1), serum (0.05 mL) from a vaccinated or control bird was added, and the sample was serially two-fold diluted in each subsequent well across the plate. This was followed by the addition of 1% sRBC solution (0.05 mL) and incubation of the plate at 37 °C for 20 min. The highest dilution of serum resulting in complete HA was recorded as the HA titer.

Humoral response against the IBV vaccine: Humoral immune responses against the IBV vaccine (*n* = 6/group; except the negative control and positive control, with *n* = 3/group) were detected using a commercial IBV IDEXX ELISA kit following the manufacturer’s instructions.

#### Flow cytometry analysis

The PBMCs (n = 3/group) collected from the experimental birds were analyzed by flow cytometry for CD4+ and CD8+ T cell subsets at 21 dpi. For analysis, 2 × 10^5^ cells were stained with anti-chicken CD4/CD8 R-PE and CD3-FITC labeled mouse monoclonal antibodies (Abcam, USA) and held overnight at 4 °C in the dark. Subsequently, the cells were washed with PBS containing 2% fetal bovine serum, and aliquots of 1.0 × 10^4^ cells were analyzed using BD FACS Calibur instrument (BD BioSciences, UK). Unstained cells served as negative controls. The CD3+ cells were gated in the FL-1 channel, which included both CD4+ and CD8+ T cells. Further, the percent of CD4+ or CD8+ cells was determined by FL-2 gating.

#### *IFN-γ* expression analysis

Concanavalin A (Con A) induced *IFN-γ* expression in PBMCs (*n* = 6/group) was evaluated at 21 dpi to determine T cell function in the experimental birds. Blood was collected in heparinized vials containing 20 IU/mL, and PBMCs were isolated and re-suspended in RPMI-1640 complete medium containing 2% fetal bovine serum, 100 IU/mL penicillin, and 100 μg/mL streptomycin. Cells were stimulated in the presence or absence of Con A (15 μg/mL) (Sigma, MO, USA) and were harvested at 12 and 24 h post-stimulation. Isolation of total RNA and synthesis of cDNA were performed as described earlier. Real time PCR was used to assess *IFN-γ* expression as outlined earlier.

#### Macrophage function test

PBMCs were isolated from experimental birds at 21 dpi (*n* = 6/group) and then stimulated *in vitro* with 10 μg/mL lipopolysaccharide (LPS; Sigma, MO, USA) for 24 h. Production of NO was assayed using Griess reagent as described earlier. The difference between NO production in stimulated and unstimulated cells from the same bird was calculated.

#### Histopathology of bursae

Sample collection and storage: Bursae (*n* = 3/group) were collected from the birds at 5 dpi and fixed in 10% neutral buffered formal saline for a minimum of 48 h at room temperature.

Histopathology: Bursae were processed in the Central Histopathology Laboratory, Indian Veterinary Research Institute. Briefly, tissues were dehydrated in ascending grades of alcohol, cleared in xylene and embedded in paraffin. Sections were cut to a thickness of 5-μm and stained with hematoxylin and eosin^25^.

Scoring bursal lesions: Hematoxylin and eosin-stained sections of bursae were microscopically examined and bursal lesion scores (BLS) were assigned as reported elsewhere^26^. Briefly, BLS were recorded on a scale of 0–4, by a blinded assessment. Scores indicated the following: 0, apparently normal lymphoid follicles; 1, mild lymphoid depletion because of a reduction in the lymphocyte population, without any sign of focal necrosis or remarkable edema; 2, moderate lymphoid depletion, along with focal necrosis and interfollicular edema; 3, severe lymphoid depletion with almost no lymphocytes and only reticular cells and proliferating fibrous tissue remaining; and 4, atrophy of follicles usually with cystic spaces, in-folding of the epithelium, and marked fibroplasia.

#### Bursa to body weight ratio

Five days after IBDV vaccination, bursa to body weight (B/B) ratio was calculated as (bursa weight/body weight) × 1000^27^.

### Weight gain in experimental birds

The body weight of each experimental bird (n = 6/group) was determined on the day of vaccination and at 21 dpi. The difference in weights between the two periods was considered the weight gained.

### Statistical analysis

Three birds per group were sacrificed to assess bursal damage at 5 dpi with seven chickens in each treatment-group for rest of the experiment. Six birds per-group were sampled in each of the experiments, except flow cytometry, where it was *n* = 3/group. Stimulation of PBMCs with TLR agonist(s) *in vitro* was analyzed by two-way analysis of variance (ANOVA) to find the effect of the treatment (TLR agonist), as well as the effect of time. Tukey’s test served as a *post-hoc* method to compare the pairwise mean difference.

To detect possible synergistic effects, fold changes in immune response genes in the Pam3CSK4 group were added to the values in the poly I:C group at each time point to generate the expected values. These composite values were compared with observed values in the Pam3CSK4 plus poly I:C groups using independent t test. Similarly, the synergistic effect was analyzed for NO production. Minimum level of significance were set at 95%, and multiplicity adjusted *P* values were calculated to minimize the alpha error. Data from the immunization study were analyzed by one-way ANOVA with Tukey’s *post-hoc* test. Mean differences were considered significant when *P* ≤ 0.05. GraphPad Prism 7.0 was used for the statistical analyses.

## Results

### Pam3CSK4 and poly I:C combination synergistically upregulated type I interferon and Th1 and Th2 cytokine transcripts in chicken PBMCs

Activation of TLRs leads to the production of specific cytokines in host cells that determine the nature of the adaptive immune responses that follows^28^. Accordingly, we analyzed the effect of Pam3CSK4 and/or poly I:C on cytokine gene expression in chicken PBMCs *in vitro* and estimated NO production at 24 and 48 h post-stimulation.

**Figure 1:**
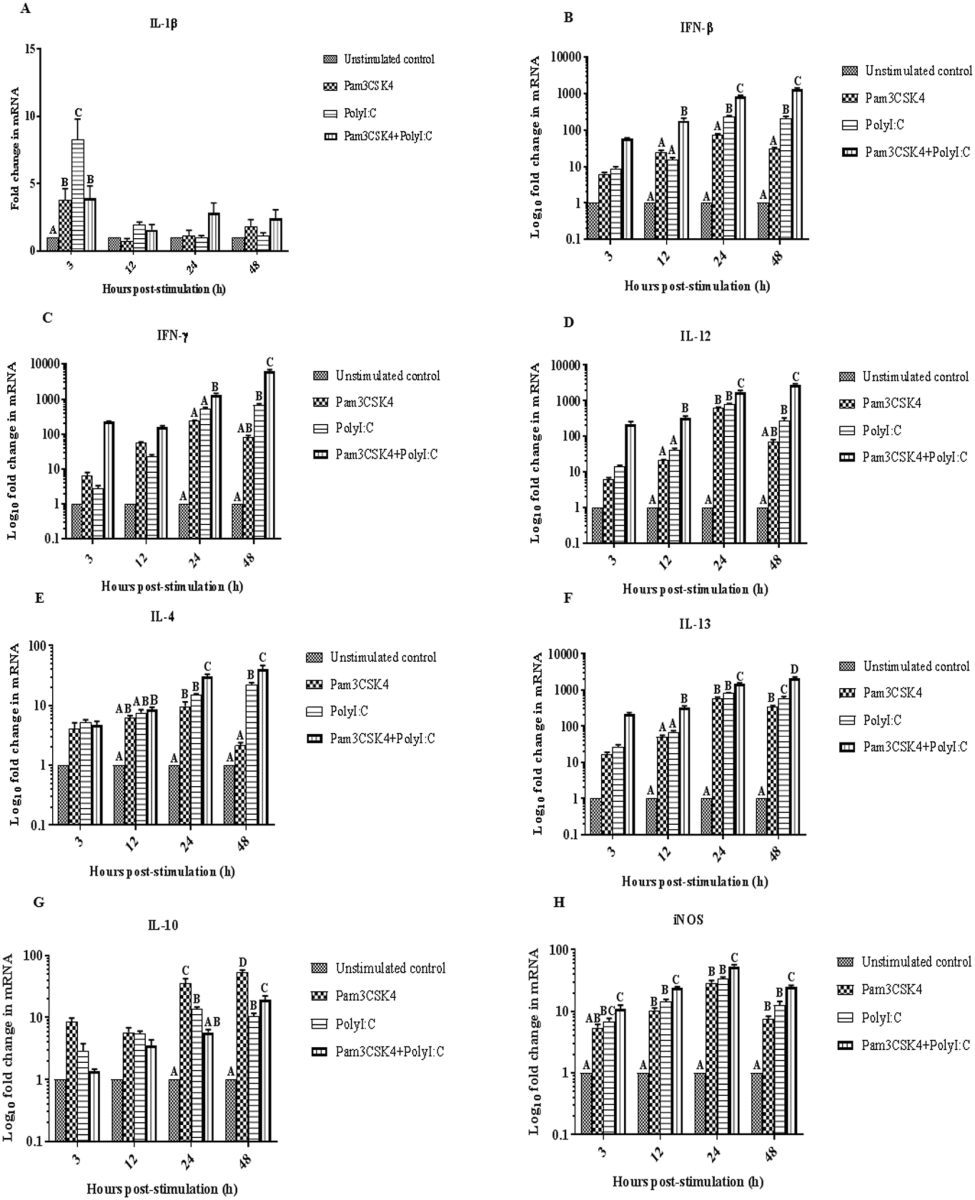
(**A**–**H**) Relative expression of immune response gene transcripts in chicken peripheral blood mononuclear cells (PBMCs) stimulated with Pam3CSK4 (10 μg/ml) and/or poly I:C (50 μg/ml) for 48 h. A two-way analysis of variance was performed to determine the effects of treatment and time (*n* = 6/treatment). The Tukey test served as a *post-hoc* test. Multiplicity adjusted *P* values were calculated to minimize the alpha error (*P* < 0.05). Different superscripts above bars (mean ± SE) indicate significant effects of Toll-like receptor agonist(s) at that time point. The experiment was repeated twice and the results from the first experiment were taken for analysis.

Pam3CSK4 and poly I:C in combination synergistically upregulated the expression of Th1 (*IL-12* and *IFN-γ*) and Th2 (*IL-4* and *IL-13*) cytokine transcripts as well as *IFN-β* transcripts, and cross-inhibited *IL-1β* and *IL-10* transcript expression (Fig. 1A–H). The peak levels of *IFN-β*, *IFN-γ*, *IL-12*, *IL-4* and *IL-13* transcripts were observed at 48 h post stimulation; these levels were 1346.67 ± 86.37, 6268.81 ± 706.50, 2855.55 ± 148.18, 40.80 ± 6.01 and 2110.10 ± 168.66 fold, respectively. In contrast, the highest transcript expression levels of *IL-1β* (3.93 ± 0.90 fold) and *iNOS* (53.91 ± 3.03 fold) were observed at 3 and 24 h post stimulation, respectively.

Individually, Pam3CSK4 and poly I:C each upregulated the expression of *IL-1β*, *IFN-β*, *IFN-γ*, *IL-12*, *IL-4*, *IL-13*, *IL-10* and *iNOS* transcripts in chicken PBMCs (Fig. 1A–H). With Pam3CSK4, peak expression levels of all genes were observed at 24 h post stimulation, except for *IL-1β* and *IL-10*, which showed the highest expression at 3 and 48 h post-stimulation, respectively. The peak levels of various transcripts following Pam3CSK4 stimulation were 3.78 ± 0.85, 72.60 ± 5.44, 240.68 ± 9.69, 621.25 ± 39.12, 9.69 ± 1.75, 604.07 ± 27.35, 44.53 ± 9.93, and 29.16 ± 2.52 fold, for *IL-1β*, *IFN-β*, *IFN-γ*, *IL-12*, *IL-4*, *IL-13*, *IL-10* and *iNOS*, respectively. In the case of poly I:C, the highest expression of *IL-1β* was observed at 3 h post-stimulation, whereas fold changes in the expression of *IFN-β*, *IL-12*, *IL-10* and *iNOS* reached their maximum at 24 h post stimulation. The peak values for *IFN-γ, IL-4* and *IL-13* transcripts were observed at 48 h after poly I:C stimulation. The highest levels of *IL-1 β*, *IFN-β*, *IFN-γ*, *IL-12*, *IL-4*, *IL-13*, *IL-10* and *iNOS* transcripts following poly I:C stimulation were 8.30 ± 1.50, 229.58 ± 17.50, 160.75 ± 65.62, 786.72 ± 47.99, 22.25 ± 1.87, 585.80 ± 75.64, 13.70 ± 1.09, and 33.74 ± 2.19 fold, respectively.

**Figure 2:**
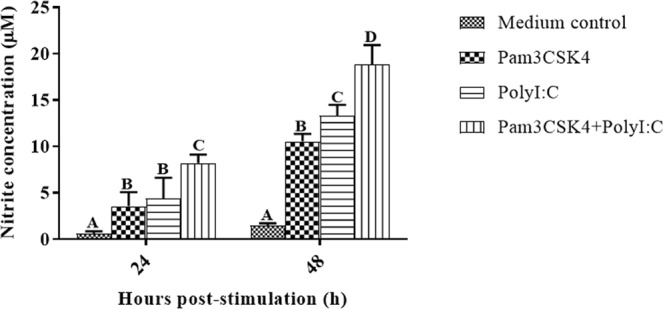
Production of nitric oxide (NO; μM) by chicken peripheral blood mononuclear cells (PBMCs) stimulated with Pam3CSK4 (10 μg/ml) and/or poly I:C (50 μg/ml) over a period of 48 h (*n* = 6/treatment). A two-way analysis of variance was performed to determine the effects of treatment and time. The Tukey served as a *post-hoc* test. Multiplicity adjusted *P* values were calculated to minimize the alpha error (*P* < 0.05). Different uppercase letters (**A**–**D**) above bars (mean ± SE) indicate significant effects of Toll-like receptor agonist at that time point.

Pam3CSK4 and/or poly I:C induced significantly higher levels of NO than those in PBMCs of unstimulated control at each time point studied (Fig. 2). Pam3CSK4 was less effective in inducing NO production at 48 h post stimulation than poly I:C (*P* = 0.011) or Pam3CSK4 plus poly I:C (*P* < 0.001) treatment. Pam3CSK4 and poly I:C in combination significantly stimulated NO production at 24 h post-stimulation when compared to the individual effects of either agonist (*P* < 0.001). Conversely, the combination showed greater cross-inhibition (*P* = 0.0019) of NO production at 48 h of incubation than that of the effects of the two agonists added. The concentrations of NO following treatment with Pam3CSK4, poly I:C and the two agonists combined reached their peaks at 48 h post-stimulation; these concentrations were 10.54 ± 0.34, 13.33 ± 0.47 and 18.84 ± 0.85 μM, respectively.

### Pam3CSK4 and poly I:C in combination reversed hot IBDV vaccine-induced immunosuppression

**Figure 3:**
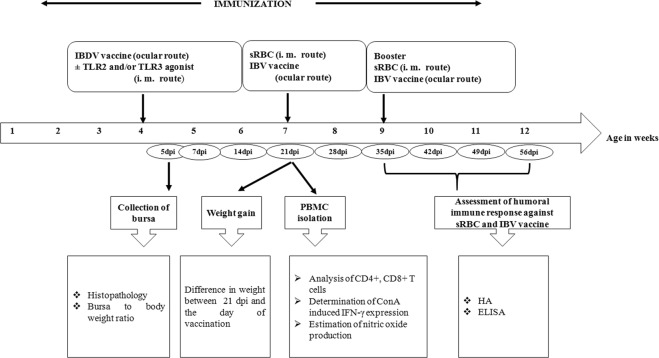
Schematic diagram showing the experimental design. IBDV: infectious bursal disease virus, sRBC: sheep erythrocytes, IBV: infectious bronchitis virus, dpi: days post-IBDV immunization, HA: hemagglutination test, ELISA: enzyme-linked immunosorbent assay.

IBDV primarily infects immature B cells and has an indirect effect on cell mediated immunity^11^, which leads to poor immune responses to other antigens or vaccines and to immunosuppression. The potential for Pam3CSK4 with or without poly I:C to alleviate deleterious effects induced by hot IBDV vaccination was assessed (Fig. 3) by an evaluation of humoral immune responses against sRBCs and the IBV vaccine, and analyses of T cell subsets, T-cell functioning, macrophage functioning, bursa to body weight (B/B) ratio and histopathology of bursae. The experimental birds were apparently normal and did not show apathy or any adverse effects after administration of TLR agonist (s).

**Figure 4:**
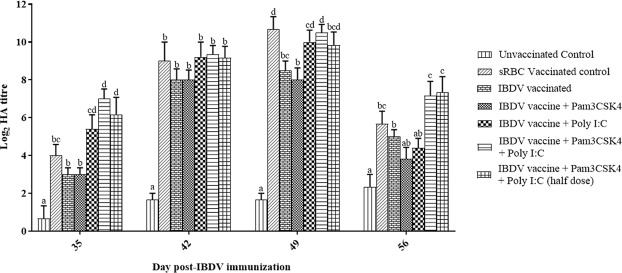
Hemagglutination (HA) titers in specific pathogen-free chickens in response to sheep red blood cells (sRBC). Birds were immunized at 4 weeks of age by ocular instillation of live intermediate plus IBDV vaccine and intramuscular injection of Pam3CSK4 and/or poly I:C. All of the birds (*n* = 7/group) were immunized with sRBCs (10% v/v at a dose rate of 1 ml/bird, intramuscular route) at 21 dpi, with a booster administered 14 days later. The positive control included three birds receiving sRBCs without prior IBDV vaccination, and negative control birds (*n* = 3) received PBS through the intramuscular route. Humoral immune responses against sRBCs (*n* = 6/group; except for the negative control and positive control groups, with *n* = 3/group) were measured by HA testing at weekly intervals until 56 dpi. Treatment effects were analyzed by one-way analysis of variance. The probability of an alpha error was set at 0.05. Different superscripts above bars (mean ± SE) indicate statistically significant differences (*P* < 0.05). dpi: days post-IBDV immunization.

**Figure 5:**
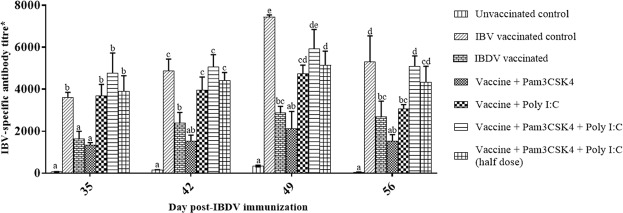
Infectious bronchitis virus (IBV)-specific antibody titers in specific pathogen-free chickens. Birds were immunized at 4 weeks of age by ocular instillation of live intermediate plus IBDV vaccine and intramuscular injection of Pam3CSK4 and/or poly I:C. All the birds (*n* = 7/group) were immunized with live IBV vaccine through ocular instillation at 21 dpi, with a booster given 14 days later. The positive control group included three birds receiving the IBV vaccine strain without prior IBDV vaccination whereas birds in the negative control group (*n* = 3) were administered PBS through the intramuscular route. Humoral immune responses against IBV (*n* = 6/group; except for the negative and positive control groups, with *n* = 3/group) were measured using a commercial IDEXX ELISA kit at weekly intervals until 56 dpi. Treatment effects were analyzed by one-way analysis of variance. The probability of an alpha error was set at 0.05. Different superscripts above bars (mean ± SE) indicate statistically significant differences (*P* < 0.05). dpi: days post-IBDV immunization. *IBV specific antibody titers greater than 396 were considered positive responses as recommended by the ELISA kit.

Antibody responses against sRBCs and the IBV vaccine in the experimental birds were analyzed 3-weeks post-IBDV vaccination. Chickens in each of the TLR agonist(s) treatment groups, except for Pam3CSK4 alone, showed significantly (*P* < 0.05) higher HA titers in response to sRBCs than to the IBDV vaccine alone at 35 dpi. Chickens in the combination treatment group showed an HA titer of 7.00 ± 0.52, whereas that for the vaccine only group was 3.00 ± 0.36 at 35 dpi. Chickens in the vaccine only (8.50 ± 0.50) and Pam3CSK4 only (8.00 ± 0.63) groups showed significantly lower (*P* < 0.05) HA titers than those in the sRBC positive control group (10.67 ± 0.67) at 49 dpi (Fig. 4). IBV-specific antibody responses were evaluated using commercial ELISA kit. The birds treated with poly I:C or poly I:C in combination with Pam3CSK4 showed significantly higher IBV-specific antibody responses than those in the vaccine and Pam3CSK4 only groups at 35 and 42 dpi (*P* < 0.05). The average titer at 35 dpi in chickens treated with the combination was 4768.69 ± 955.25, as compared to 1638.51 ± 353.33 for chickens in the IBDV vaccine only group. There was a significantly lower (*P* < 0.05) IBV-specific antibody response in the birds treated with the vaccine or Pam3CSK4 alone than in the IBV-positive control birds at all time points studied (Fig. 5).

**Figure 6:**
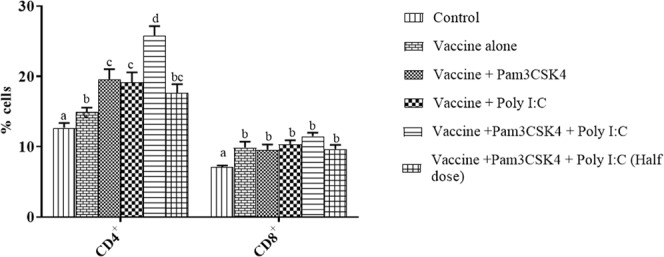
Percentages of CD4^+^ and CD8^+^ T cells in peripheral blood mononuclear cells (PBMCs) collected from specific pathogen-free chickens. Birds were immunized at 4 weeks of age by ocular instillation of live intermediate plus IBDV vaccine and intramuscular injection of Pam3CSK4 and/or poly I:C. PBMCs (*n* = 3/group) were analyzed by flow cytometry to assess the T cell subsets at 21 dpi using the chicken specific fluorescently labelled monoclonal antibodies. Treatment effects were analyzed by one-way analysis of variance. The probability of an alpha error was set at 0.05. Different superscripts above bars (mean ± SE) indicate statistically significant differences (*P* < 0.05). dpi: days post IBDV immunization.

We determined the percentage of CD4+ and CD8+ T cells at 21 dpi by flow cytometry. The vaccinated birds with or without TLR agonist(s) showed significantly higher levels of CD4^+^ and CD8^+^ T cells than those in the control group birds at 21 dpi (*P* < 0.05). Both CD4^+^ and CD8^+^ T cell numbers were highest in the combination-treated birds (Fig. 6), which showed values of 25.83 ± 1.33% and 11.50 ± 0.50%, respectively.

**Figure 7:**
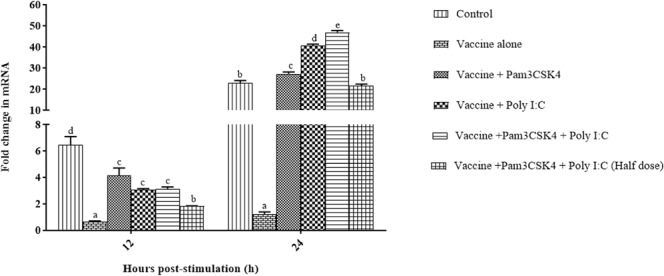
Expression of *IFN-γ* transcripts in peripheral blood mononuclear cells (PBMCs) isolated from specific pathogen-free chickens and stimulated with ConA. Birds were immunized at 4 weeks of age by ocular instillation of live intermediate plus IBDV vaccine and intramuscular injection of Pam3CSK4 and/or poly I:C. PBMCs (*n* = 6/group) were isolated and stimulated with Con A (15 μg/ml) keeping medium control. Expression of *IFN-γ* transcripts was analyzed by real-time PCR at 12 and 24 h post-stimulation. Treatment effects were analyzed by one-way analysis of variance. The probability of an alpha error was set at 0.05. Different superscripts above bars (mean ± SE) indicate statistically significant differences (*P* < 0.05). dpi: days post-IBDV immunization.

T cell function in the experimental birds was assessed by determining Con A-induced *IFN-γ* expression in PBMCs at 21 dpi. In comparison with birds in other groups, there was significantly (*P* < 0.05) lower levels of *IFN-γ* expression in birds of the vaccine only group at both 12 h (0.65 ± 0.06) and 24 h (1.21 ± 0.18) post-stimulation (Fig. 7). In the combination treatment group, the *IFN-γ* transcript levels were 3.12 ± 0.17 and 46.97 ± 0.88 fold upregulated at 12 and 24 h post-stimulation, respectively. The TLR agonist(s)-treated birds (groups C, D, and E) showed significant upregulation of *IFN-γ* (*P* < 0.05) compared to those in the unvaccinated control (group A) at 24 h post-stimulation.

**Figure 8:**
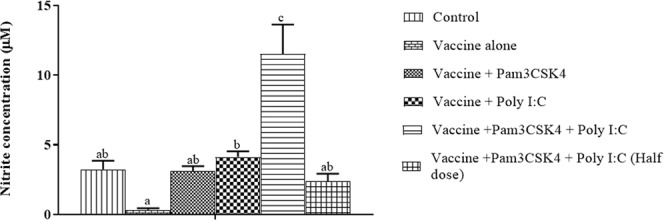
Differential nitric oxide (NO) production by peripheral blood mononuclear cells (PBMCs) isolated from specific pathogen-free chickens and stimulated with LPS. Birds were immunized at 4 weeks of age by ocular instillation of live intermediate plus IBDV vaccine and intramuscular injection of Pam3CSK4 and/or poly I:C. PBMCs (*n* = 6/group) were isolated on 21 dpi and stimulated using LPS (10 μg/ml) keeping medium control. The production of NO was analyzed using Griess reagent as a nitrite at 24 h post-stimulation, and the difference between stimulated and unstimulated cells in the same bird was calculated. Treatment effects were analyzed by one-was analysis of variance. The probability of an alpha error was set at 0.05. Different superscripts above bars (mean ± SE) indicate statistically significant differences (*P* < 0.05). dpi: days post-IBDV immunization.

Macrophage functioning was determined by assessing NO production in PBMCs collected from the experimental birds upon stimulation with LPS *in vitro*. NO production was significantly greater in the poly I:C only (4.13 ± 0.41 µM) and combination (11.52 ± 2.12 µM) treatment groups when compared to the vaccine only group (0.34 ± 0.11 µM). In other groups, the NO production was numerically greater than that in birds of the vaccine only group (Fig. 8).

**Figure 9:**
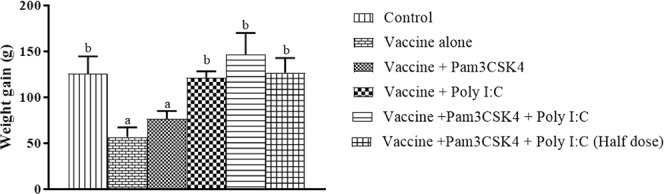
Body weight gain in specific pathogen-free chickens. Birds were immunized at 4 weeks of age by ocular instillation of live intermediate plus IBDV vaccine and intramuscular injection of Pam3CSK4 and/or poly I:C. The differences in body weights of experimental birds (*n* = 6/group) at 21 dpi and on the day of vaccination were calculated. Treatment effects were analyzed by one-way analysis of variance. The probability of an alpha error was set at 0.05. Different superscripts above bars (mean ± SE) indicate statistically significant differences (*P* < 0.05). dpi: days post-IBDV immunization.

Body weight gain was calculated as the difference in weights at 21 dpi and the day of vaccination. Weight gain in birds in the poly I:C (121.25 ± 7.03 g), combination (146.50 ± 23.64 g) and combined half dose (126.56 ± 16.27 g) treatment groups was significantly greater (P < 0.05) than that in birds in the vaccine only (56.69 ± 10.51 g) and Pam3CSK4 (76.56 ± 8.56 g) treatment groups (Fig. 9) and was comparable to that in unvaccinated control birds (125.81 ± 18.85 g).

**Figure 10:**
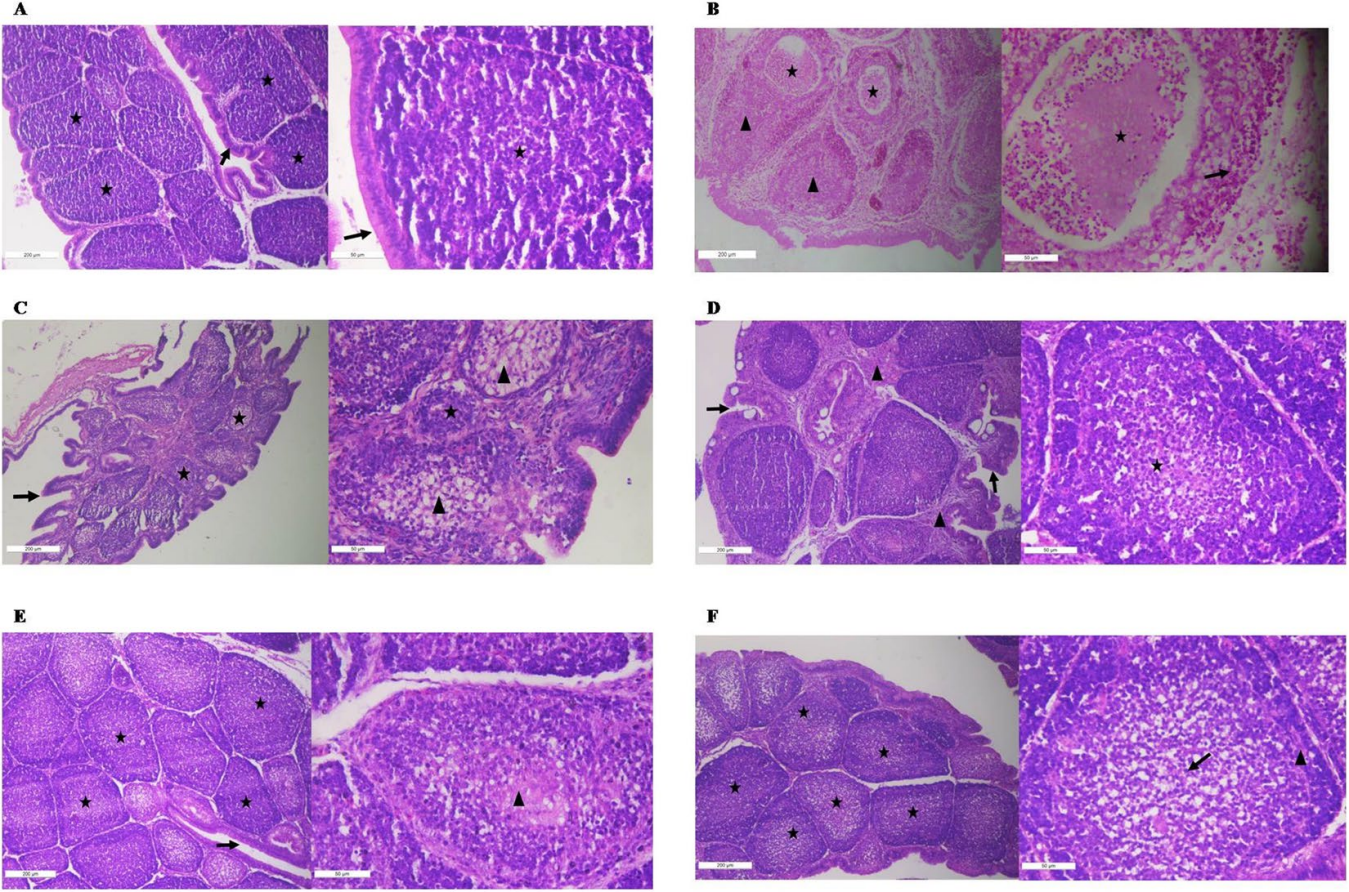
Histopathology of bursae in specific pathogen-free chickens inoculated with live intermediate plus IBDV vaccine and Pam3CSK4 and/or poly I:C. Birds were immunized at 4 weeks of age by ocular instillation of live intermediate plus IBDV vaccine and intramuscular injection of Pam3CSK4 and/or poly I:C. Bursae were collected on dpi 5. A representative photomicrograph of a 5-mm thick section of bursa stained with hematoxylin and eosin is shown. (**A**) Apparently normal histoarchitecture of bursal follicles (★) lined with intact epithelium (→) (100× and 400×) in control (non-immunized) birds. (**B**) Cystic follicle (★), lymphoid depletion (►) and thinning of the cortex (→) (grade-3; 100× and 400×) in birds immunized with IBDV vaccine alone (**C**) Atrophy of bursal follicles (★) lined with hyperplastic epithelium (→). Severe lymphoid depletion in the cortex and medulla, and severe reticuloendothelial (RE) cell hyperplasia (►) (grade-3; 100× and 400×) in birds receiving Pam3CSK4 plus IBDV vaccine. (**D**) Mild fibrosis of inter-bursal follicles (►) lined with hyperplastic epithelium (→) and intact follicles. Mild lymphoid depletion in the medulla (★) (grade-1; 100× and 400×) in poly I:C plus IBDV vaccine immunized birds. (**E**) Apparently normal histoarchitecture of bursal follicles (★) lined with intact epithelium (→). Mild lymphoid depletion in the medulla and mild RE cell hyperplasia (►) (grade-1; 100× and 400×) in full-dose Pam3CSK4 plus poly I:C IBDV vaccine immunized birds. (**F**) Intact follicles (★) and epithelial lining. Mild to moderate lymphoid depletion in the medulla (→), mild RE cell hyperplasia and thinning of the cortex (►) (grade-2) in half-dose Pam3CSK4 plus poly I:C IBDV vaccine immunized birds (100× and 400×).

**Figure 11:**
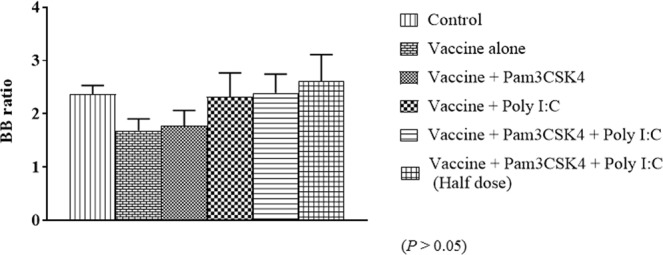
Bursa to body weight (B/B) ratio in specific pathogen-free chickens. Birds were immunized at 4 weeks of age by ocular instillation of live intermediate plus IBDV vaccine and intramuscular injection of Pam3CSK4 and/or poly I:C. Bursae (*n* = 3/group) were collected on dpi 5 and BB ratio was calculated as (bursa weight/body weight) × 1000. Treatment effects were analyzed by one-way analysis of variance. The probability of an alpha error was set at 0.05. Different superscripts above bars (mean ± SE) indicate statistically significant differences (*P* < 0.05). dpi: days post-IBDV immunization.

Since the hot IBDV vaccine can cause bursal damage, we examined the histopathology of bursae collected from the experimental birds at 5 dpi and the BLS is presented in Table 3. The bursae of control birds showed intact follicular epithelia and bursal follicles that contained lymphocytes, with a mean score of 0 as there were no visible pathological changes (Fig. 10A). Histologically, bursae from the vaccine only group showed multifocal cysts in bursal follicles, with severe lymphoid depletion and thinning of cortices. The mean bursal score for this group was 3.3 (Fig. 10B). In contrast, bursae from the Pam3CSK4 and poly I:C combination treatment group revealed normal follicular histoarchitechture, with intact epithelia, mild medullary lymphoid depletion and reticuloendothelial (RE) cell hyperplasia (Fig. 10E), resulting in a mean bursal score of 1.0. Bursae of the Pam3CSK4 group showed mild follicular atrophy with hyperplastic epithelia, moderate to severe lymphoid depletion in cortices and medullae, and severe RE cell hyperplasia (Fig. 10C). Thus, the mean bursal score for this group was 2.3. Birds in the poly I:C group revealed intact follicles surrounded by mild inter-bursal follicular fibrosis and hyperplastic epithelium. Mild lymphoid depletion was evident in medullae (Fig. 10D), resulting in a mean bursal score of 1.3 in this group. Bursae from birds in the half-dose group showed follicles with mild to moderate lymphoid depletion in the medullae, mild RE cell hyperplasia, and thinning of cortices (Fig. 10F). The mean bursal lesion score for this group was 1.6 because of moderate depletion of lymphoid cells. The B/B ratio was calculated in the humanely sacrificed birds at dpi 5. It was comparable among the groups and was highest in the Pam3CSK4 and poly I:C combination in half dose group with the value of 2.62 ± 0.49 (Fig. 11).

**Table 3:** Bursal lesion score determined by a blinded assessment in the experimental birds.

Group (*n* = 3/group)	Bursal lesion score			
	Bird 1	Bird 2	Bird 3	Average
A (PBS control)	0	0	0	0
B (Vaccine alone)	3	4	3	3.33
C (Pam3CSK4 group)	2	3	2	2.33
D (Poly I:C group)	2	1	1	1.33
E (Combination group)	1	1	1	1
F (Combination in half dose group)	2	1	2	1.67

### Pam3CSK4 and poly I:C in combination elicited IBDV-specific antibody responses that were comparable to those following IBDV vaccination alone

**Figure 12:**
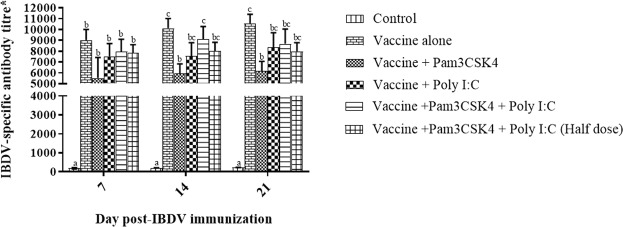
Infectious bursal disease virus (IBDV)-specific antibody titers in specific pathogen-free chickens. Birds were immunized at 4 weeks of age by ocular instillation of live intermediate plus IBDV vaccine and intramuscular injection of Pam3CSK4 and/or poly I:C. IBDV-specific antibody responses (*n* = 6/group) were monitored by testing with a commercial ELISA kit (IDEXX laboratories, USA) at weekly intervals until 21 dpi. Treatment effects were analyzed at each time point by one-way analysis of variance. The probability of an alpha error was set at 0.05. Different superscripts above bars (mean ± SE) indicate statistically significant differences (*P* < 0.05) at specific time points. *IBDV- specific antibody titers greater than 396 were considered positive responses as recommended by the ELISA kit. dpi: days post IBDV immunization.

We also evaluated the effects of Pam3CSK4 and/or poly I: C on humoral immune responses elicited by the IBDV vaccine. IBDV-specific antibody responses were evaluated using a commercial ELISA kit at weekly intervals until 21 dpi. Antibody responses in the poly I:C, combination and half dose treatment groups were comparable with those in the vaccine only group (*P* > 0.05) at each time point (Fig. 12). In contrast, birds in the Pam3CSK4 group showed significantly lower antibody titers than birds in the vaccine only group at 14 and 21 dpi (*P* < 0.05).

## Discussion

Stimulation of a single TLR rarely occurs in nature as pathogens carry multiple pathogen associated molecular patterns that stimulate multiple TLRs simultaneously. Thus, using TLR agonists in combination to modulate the host immune responses may lead to more desirable effects than using these agonists individually. The potential of such an approach is becoming evident^20,29,30^. Co-stimulation of membranes and endosomal TLRs has a synergistic effect in chickens^20,30^. Despite the fact that live intermediate plus IBDV vaccines (hot vaccines) alter immune functions by affecting B cells directly and cellular immune responses indirectly^11,31–34^, these remain the main tools for the control of IBD. Therefore, in the present study, we evaluated the potential for Pam3CSK4 and/or poly I:C to derepress IBDV vaccine-induced immunosuppression in SPF chickens. White Leghorn SPF chickens were chosen for use in the experiment as they are highly susceptible to infection with very virulent strains of IBDV^35,36^. The expression of immune response genes and NO production following stimulation of chicken PBMCs with TLR2 and/or TLR3 agonists *in vitro* was investigated to understand the mechanistic basis of derepression.

Binding of Pam3CSK4 to TLR2 activates a reaction cascade that involves the interactions between the cytoplasmic Toll/IL-1 receptor (TIR) domain of TLR2 and intracellular adapter molecules such as MyD-88 that lead to the activation of transcription factors *NF-κB* and mitogen activated protein kinases. This results in the expression of pro-inflammatory mediators^37^. In the present study, Pam3CSK4 upregulated *IL-1β*, *IFN-β*, *IFN-γ*, *IL-12*, *IL-4*, *IL-13*, *IL-10* and *iNOS* transcripts and increased NO production in the chicken PBMCs (Figs 1A–H and 2). These results are supported by the findings of earlier studies on chicken splenocytes^38,39^, cecal tonsil mononuclear cells^40^, vaginal and uterine mucosa^41^, tracheal organ culture^42^ and macrophages^43^. Administration of TLR2 ligands *in ovo* stimulated the production of *IFN-γ* and upregulated IFN stimulatory genes in chorioallantoic membranes of embryonated eggs^44^. Stimulation of TLR3 by poly I:C activates a *MyD88* independent pathway that signals through TIR-domain-containing adapter-inducing interferon-β (TRIF) leading to the activation of *IRF-3* and *NF-κB* and culminating in the production of type I IFN and inflammatory cytokines^45^. Upregulation of *IL-1β*, *IFN-β*, *IFN-γ*, *IL-12*, *IL-4*, *IL-13*, *IL-10* and *iNOS* transcripts and an increase in NO production in chicken PBMCs (Figs 1A–H, 2) upon stimulation with poly I:C agree with results observed in chicken PBMCs^46^, bursal cells^47^, and RAW 264.7 cells^48^. In addition, treatment of dendritic cells with poly I:C has been found to stimulate the secretion of *IL-12* in humans^49^ and mice^50^.

Pam3CSK4 and poly I:C, administered in combination, synergistically upregulated the expression of Th1 (*IL-12* and *IFN-γ*) and Th2 (*IL-4* and *IL-13*) cytokines, as well as *IFN-β* transcripts (Fig. 1A–H), in chicken PBMCs. This indicates that administering TLR2 and TLR3 agonists in combination efficiently induces a well-balanced Th1/Th2 immune response in chickens. Synergistic TLR-TLR cross-talk has been reported following stimulation with various TLR agonists in combination in chickens^20,30,38,46,51–53^ as well as in other species^54–58^. The synergistic upregulation of various transcripts observed in the present study might be the result of cooperation between downstream molecules in the MyD88-dependent and TRIF-dependent pathways. Similar to our results, stimulation of murine myeloid dendritic cells with Pam3CSK4 and poly I:C *in vitro* was shown to increase production of *IL-12p40* and *IFN-γ*^59^ and lipotechoic acid acted synergistically with poly I:C to boost *IL-12* response in mice^60^. The present study showed that Pam3CSK4 and poly I:C administered in combination resulted in cross-inhibition of *IL-1β*, *IL-10* and *iNOS* transcript expression and NO production (Figs 1A–H, 2) in chicken PBMCs. In contrast, the TLR2-TLR3 interactions showed synergy in the production of *IL-6* and *IL-8* in human airway epithelial cells^61^ and the production of *IL-1β*, *iNOS*, chemokine ligand (CCL)-5 and TNF in mice macrophages *in vitro*^18^. Earlier reports showed that intra-uterine administration of poly I:C and peptidoglycan synergistically induced inflammatory mediators, including *IL-1β* in mice^62^. This synergism might be the result of TLR2 upregulation by poly I:C^61^. In addition, Pam3CSK4 and poly I:C synergistically increased the survival and proliferation of B cells in mice by promoting the secretion of *IL-6* and *TNF-α*^63^. The difference between *IL-1β* expression in mice and chickens upon stimulation with Pam3CSK4 and poly I:C indicates species-specific effects.

In the present study, use of TLR agonist(s) with hot IBDV vaccines in SPF chickens was beneficial; it allowed the birds to maintain immunocompetence after IBDV vaccination. The immune status of birds after IBDV vaccination can be determined by assessing humoral responses against different antigens such as *Brucella abortus*, sRBCs, or ND virus vaccine^64–66^. Prior infection with a very virulent IBDV strain was shown to affect humoral immune responses to IBV^10^, turkey herpes virus vaccination^67^. Sheep erythrocytes are recommended by the OIE as an alternative test antigen for the assessment of immunosuppression after live IBDV vaccination^68^. Considering the clonal nature of immunity, surveying responses to a single type of vaccine or antigen might provide only partial information about immunosuppression. Here, we evaluated the ability of experimental birds to respond to both live IBV vaccine and sRBCs 3-weeks post-IBDV vaccination. Birds in the poly I:C, Pam3CSK4 plus poly I:C and Pam3CSK4 plus poly I:C (half dose) groups showed greater antibody responses to both sheep erythrocytes (Fig. 4) and the IBV (Fig. 5) than those of birds in the vaccine only group, indicating the beneficial effects of TLR agonist(s) on B cells. In some instances, birds in the Pam3CSK4 plus poly I:C and Pam3CSK4 plus poly I:C (half dose) groups showed numerically higher antibody responses to both sRBCs and IBV vaccine than those of birds in the unvaccinated control group. The results indicate that administration of poly I:C alone or in combination with Pam3CSK4 allows B cell responses to be maintained after hot IBDV vaccination.

Since IBDV adversely affects T cell function indirectly^31–33^, we evaluated both the number and function of specific T cell subsets by flow cytometry, as well as *IFN-γ* expression post-IBDV vaccination. Vaccination with intermediate plus IBDV vaccine has been shown to simultaneously increase the number of CD8^+^ T cells and decrease the number of CD4^+^ T cells^69,70^. In the present study, we observed a significant increase in CD4^+^ T cells in the combination treated birds (Fig. 6), which supports results in the mice administered L-pampo, a proprietary adjuvant composed of Pam3CSK4 and poly I:C^71^. In addition, significant upregulation of *IFN-γ* was recorded in all of the TLR agonist(s) treated birds (Fig. 7), but not in birds in vaccine only group. This indicates the role of TLR agonists in maintaining a normal functioning T cell population as reported earlier^72^.

Infection with IBDV decreased the number and phagocytic activity of monocytes/macrophages, with lesser sensitivity to chemokines in the chickens^11,34^. In the present study, macrophage function was tested 3-weeks post IBDV immunization based on NO production. Birds in the vaccine only group showed low NO concentrations following stimulation, indicating impaired macrophage function in response to hot IBDV vaccination. TLR agonist(s) prevented IBDV-induced impairment of macrophage function, as evidenced by greater NO production by PBMCs upon stimulation with LPS *in vitro* (Fig. 8).

The target cells for IBDV are IgM-bearing B lymphocytes^73,74^. IBDV replication leads to extensive lymphoid cell depletion in the bursa of Fabricius and results in varying degrees of bursal atrophy^75,76^. Live IBDV vaccine strains also induce bursal atrophy to varying degrees^77,78^. In our study, histopathology of bursae from birds in the vaccine only group at 5 dpi showed vacuolation and severe lymphoid depletion (Fig. 10B), which agrees with earlier reports^8–10,79^. In contrast to bursae from chickens in the Pam3CSK4 group, bursae from chickens in the poly I:C, Pam3CSK4 plus poly I:C and Pam3CSK4 plus poly I:C (half dose) groups showed normal bursal histoarchitecture, with mild to moderate lymphoid depletion (Fig. 10D–F), which was represented by bursal lesion scores. These results indicate that poly I:C alone and in combination with Pam3CSK4 protected the birds from vaccine induced bursal damage. This protection could be attributed to the upregulation of *IFN-β* and balance of Th1 and Th2 immune responses shown by *in vitro* testing. Indeed, recombinant chicken IFN-β has been shown to reduce the severity of pathological lesions in bursae caused by IBDV infection^80^. Despite the reduction in the bursal lesion score, B/B ratio showed only numerical increase in the groups, D, E and F as compared to Group B (Fig. 11).

Decreased poultry production due to poor weight gain and resulting economic losses are the major impacts of IBDV infection^6,81^. Except for birds in the Pam3CSK4 group, those in the other agonist(s) treatment groups (Groups: D, E and F) showed significantly greater body weight gain (Fig. 9) than that of birds in the vaccine only group. This could be attributed to the partial restoration of immunocompetence by poly I:C alone or in combination with Pam3CSK4 after hot IBDV vaccination. Cross-inhibition of *IL-1β*, *IL-10* and *iNOS* transcript expression with concurrent upregulation of *IFN-β*, *IFN-γ*, *IL-12*, *IL-4* and *IL-13* transcripts by TLR agonists in combination can create a favorable environment for immune cells, as reported earlier^71^. Downregulation of TLR2 following IBDV infection might have reduced the effects of Pam3CSK4. In contrast, the beneficial effects of poly I:C alone might be the result of TLR3 upregulation after IBDV infection^82^.

Humoral immunity plays an important role in protection against IBDV, with a positive correlation between neutralizing antibody titers and protection demonstrated^83^. The TLR agonist(s), when administered with the hot IBDV vaccine, did not enhance IBDV-specific antibody responses significantly in the experimental birds relative to responses to the vaccine alone (Fig. 12). In earlier studies, the induction of humoral immunity following vaccination with a commercially available intermediate IBDV vaccine clearly correlated with the induction of bursal lesions and IBDV replication^78,84^. Thus, comparable immune responses against IBDV vaccine in the TLR agonist treated birds might be the result of decreased replication of the vaccine virus caused by upregulation of type I IFNs by the TLR agonist(s), which are antiviral in nature. Inhibition of IBDV replication by *IFN-β* in chickens was reported previously^80^. A decrease in avian influenza virus replication *in vitro* in a chicken macrophage cell line (MQ-NCSU) following administration of Pam3CSK4, LPS, and CpG oligonucleotide^43^ supports this notion. Similarly, poly I:C was shown to reduce avian influenza virus shedding *in vivo*^85^ and to inhibit ND virus replication in HeLa cells and DF-1 chicken fibroblast cells^86^. Agonists of TLR3, 5, 7 and 9 inhibited the replication of hepatitis B virus in mice hepatocytes^87^. In contrast, L-pampo resulted in an enhanced antibody response when administered with hepatitis B virus surface antigen in mice^88^. This difference could be because we used a live virus vaccine in our study, whereas Yum and his co-workers immunized birds with a protein antigen vaccine. Intermediate plus strain of IBDV vaccines stimulate immunity in the birds even in the presence of maternally derived antibodies^7^. As there was no significant reduction in IBDV specific antibody titer in the birds receiving poly I:C and its combination with pam3CSK4 in comparison to the vaccine alone group (Group B), it is likely that adjuvanting IBD vaccine with TLR3 or combination of TLR2 and TLR3 agonists would break through the higher maternal antibody titres. In the present study, the TLR agonist (s) was administered through intramuscular route, which is laborious and time consuming. Thus, further research is needed for evaluating the efficiency of these agonists by other routes convenient for mass vaccination like administration through drinking water or spraying. In addition, within a span of 3 to 4 days no other live viral or vectored vaccines are to be administered as these TLR agonist (s) induce an antiviral effect in the birds.

In conclusion, administration of poly I:C alone or in combination with Pam3CSK4 reduced the deleterious effects of hot IBDV vaccination by decreasing the bursal damage and B cell destruction, restoring T cell and macrophage functions and increasing weight gain to maintain immunocompetence in chickens.

## Data Availability

The datasets used or analyzed during the current study are available from the corresponding author on reasonable request.
